# First Isolation and Characterization of *Chryseobacterium cucumeris* SKNUCL01, Isolated from Diseased Pond loach (*Misgurnus anguillicaudatus*) in Korea

**DOI:** 10.3390/pathogens9050397

**Published:** 2020-05-21

**Authors:** Sang Guen Kim, Sib Sankar Giri, Sang Wha Kim, Jun Kwon, Sung Bin Lee, Se Chang Park

**Affiliations:** Laboratory of Aquatic Biomedicine, College of Veterinary Medicine and Research Institute for Veterinary Science, Seoul National University, Seoul 08826, Korea; imagine0518@snu.ac.kr (S.G.K.); giribiotek@gmail.com (S.G.G.); kasey.kim90@gmail.com (S.W.K.); kjun1002@snu.ac.kr (J.K.); lsbin1129@naver.com (S.B.L.)

**Keywords:** loach, opportunistic pathogen, antibiotic resistance, β -lactamase, efflux pump

## Abstract

Loaches are widely distributed throughout the natural environment and are consumed for medicinal purposes in East Asia. Usually, loaches are cultured in ponds where the water conditions can easily cause bacterial infections. Infections due to bacterial pathogens such as *Aeromonas* have been well described in cultured loaches; however, there is no report regarding *Chryseobacterium* infection. This study focused on the elucidation of the pathogenic and antibiotic resistance characteristics of *C. cucumeris*, SKNUCL01, isolated from diseased loaches (*Misgurnus anguillicaudatus*). SKNUCL01 forms a biofilm, which is associated with its virulence. Koch’s postulates were satisfied with a lethal dose 50 (LD_50_) of 8.52 × 10^7^ colony-forming units (CFU)/ml. Abrasion facilitates the mortality of the fish, which makes it a possible infection route for *C. cucumeris*. The strain showed resistance to nearly all tested antibiotics, such as trimethoprim/sulfamethoxazole, levofloxacin, and ciprofloxacin, formerly considered effective treatments. Phenotypic analyses for antibiotic resistance—the combined disk test, double-disk synergy test, modified Hodge test, and efflux pump inhibition test—revealed that the resistance of SKNUCL01 originated from metallo-beta lactamases (MBLs) and efflux pumps. Our findings provide evidence that could result in a breakthrough against multidrug-resistant *Chryseobacterium* infection in the aquaculture industry; the antibiotic resistance-related genes can be elucidated through future study.

## 1. Introduction

Loaches (*Misgurnus* spp.) are widely distributed throughout natural freshwater environments such as rice paddy fields, streams, and reservoirs in Korea, China, and Japan [1,2,3]. Being rich in vitamins, proteins, and taurine, loaches are commonly referred to as “ginseng in the water” and are consumed for their nourishing and tonifying properties [4,5]. For these reasons, pond loaches (*Misgurnus anguillicaudatus*) are widely cultured in Korea and China as a food animal, yielding over 400,000 tons per year, which is 300% higher than the yield of gilthead seabream (*Sparus aurata*) and is comparable to that of the well-known channel catfish (*Ictalurus punctatus*) (Food and Agriculture Organization of the United Nations, 2016). 

Usually, loaches are cultured in ponds, and the culture is often plagued by difficulties in controlling the water quality and stocking density. Such conditions can easily stress the fish, making them more vulnerable to various bacterial infections. Previously, infections with common bacterial pathogens such as *Aeromonas, Flavobacterium*, and *Vibrio* have been described in cultured loaches [6,7,8,9]. However, there is no report regarding *Chryseobacterium* infection in loaches, to the best of our knowledge. Recently, there has been an increase in clinical cases associated with isolates of *Chryseobacterium* spp. from different fish species. For instance, *C. arthori* was recovered from the kidney of pufferfish (*Arothron hispidus*) [10] and *C. shigense* was isolated from the liver, gill, and kidney of rainbow trout (*Oncorhynchus mykiss*) [11]. More recently, the pathogenicity of *C. scophthalmum* isolated from golden mahseer (*Tor putitora*) was confirmed [12]. Thus, the *Chryseobacterium* species are considered potential emerging pathogens in fish [13]. 

Chryseobacterium is notorious for multidrug resistance. In human cases, it has shown resistance against clinically important antibiotics, including cephalosporins and carbapenems [14,15,16]. There have been reports of treatment failures due to antibiotic resistance, leading to deaths [17,18]. The phenotypic antibiotic susceptibility testing and genotyping of the antibiotic resistance of the bacteria have also been well elucidated. However, only a few studies are available on the antibiotic susceptibility pattern of Chryseobacterium sp. isolated from diseased fish or associated environments [19,20,21]. In this study, we isolated C. cucumeris SKNUCL01 from diseased moribund loaches (M. anguillicaudatus). The pathogenicity and biofilm-forming capacity of the isolate were examined, and the antibiotic resistance mechanisms were inferred through phenotypic antibiotic susceptibility testing with or without specific inhibitors.

## 2. Results

### 2.1. Isolation of Bacteria SKNUCL01

A collection of fish, which included diseased fish with gross lesions on their skin, were brought to our aquatic animal facility. The animals were immersed in oxytetracycline hydrochloride (50 mg/L) for 5 days, without a positive result. This was followed by erythromycin (25 mg/L) treatment, but they failed to recover and eventually died. However, no mortality or morbidity was recorded in loaches without skin lesions. Only yellowish colonies were observed, except on skin samples that showed mixed colonies with dominant yellowish colonies. We isolated three bacterial strains from the post-mortem loaches; these were shiny, round, and yellowish colonies on tryptic soy agar (TSA) and produced a distinct odor. The straight short rod-shaped bacteria were negative to Gram staining.

### 2.2. Histological Analysis of the Skin 

**Figure 1:**
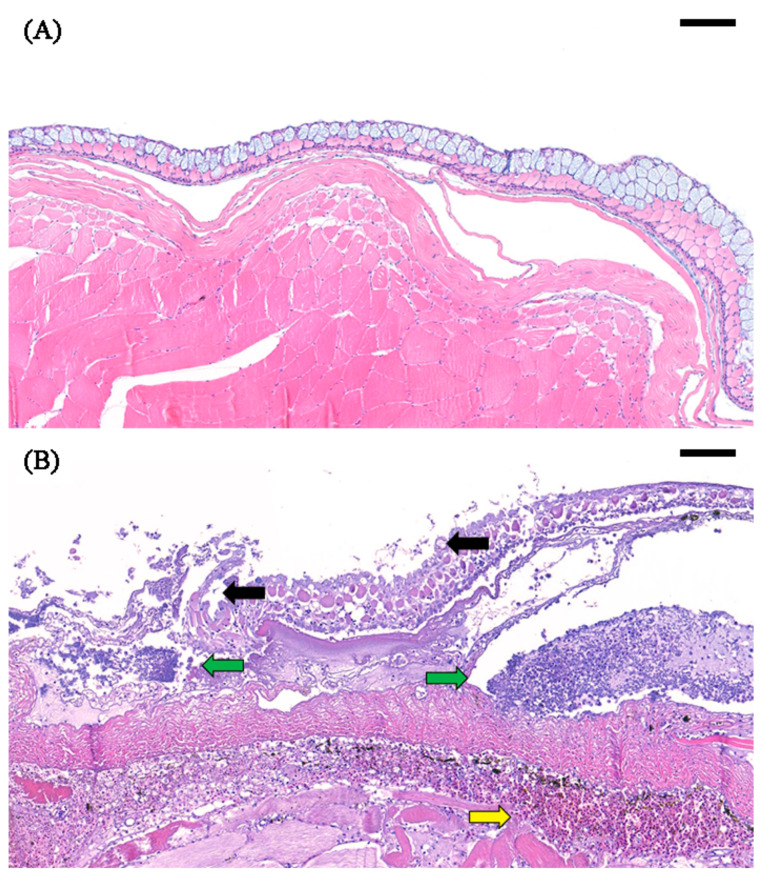
Histological analysis of the skin of a pond loach. (**A**) Normal skin with an intact epidermidis. (**B**) Infected skin with exfoliation of the epidermidis (black arrow), infiltration of inflammatory cells (green arrow), and hemorrhage (yellow arrow). Bar, 100 μm.

The histology of the skin of the naturally infected fish was examined and showed definite clinical signs, such as ulceration, loss of epidermis, and fungus-like white patches. A comparison was performed between normal skin and infected skin. The non-infected skin showed an intact epidermis and no signs of infiltration of inflammatory cells or red blood cells (Figure 1A), whereas epidermal exfoliation, damaged epithelial and club cells, inflammatory cell infiltration, and hemorrhage in the underlying dermal loose connective tissue were observed in the infected skin lesions (Figure 1B).

### 2.3. Identification of Bacteria SKNUCL01

The 16S rRNA gene sequencing revealed that the isolates shared 100% sequence homology and belonged to *C. cucumeris*. Although 16S rRNA sequence comparisons using nucleotide basic local alignment search tool (BLASTn) could not specify the species in the genus *Chryseobacterium*, the sequence matched best with *C. cucumeris* (GSE06T) both in BLASTn and EZtaxon. Thus, the biochemical characteristics, using the VITEK 2 system (bioMérieux, France), were analyzed using closely related species: *C. cucumeris* (GSE06T), *C. gleum* (F93T), and *C. indologense* (RH 542T; Table S1). All three isolates showed the same pattern, and all the indices were homologous to *C. cucumeris*, whereas the isolates showed only 93% (44/47) and 87% (41/47) homology of biochemical indices with *C. gleum*, and *C. indologense*, respectively. As glucose non-fermenting bacteria, all *Chryseobacterium* showed negative at D-glucose. The main discrimination points between *C. cucumeris* and *C. gleum* were β-xylosidase, malonate, and glycline arylamidase. However, compared to *C. indologense*, tests for L-pyrrolydonyl-arylamidase, β-glucosidase, L-proline arylamidase, lipase, succinate alkalinization, and glycine arylamidase activities showed opposite results in *C. cucumeris*. 

**Figure 2:**
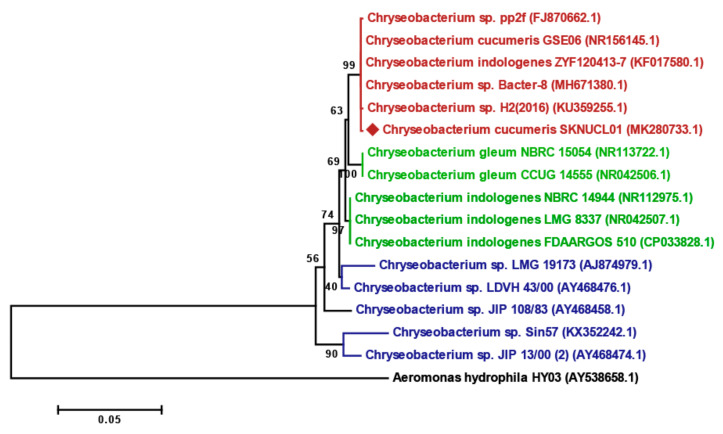
The phylogenetic tree based on partial 16S rRNA gene sequences, showing the relationship between *C. cucumeris* SKNUCL01 and related species in the genus *Chryseobacterium*. Bootstrap value based on a maximum-likelihood analysis of 1000 resamples. The sequence of *Aeromonas hydrophila* HY03 (AY538658.1) was used as an outgroup. Bar, 0.05 nucleotide substitutions per site.

The phylogeny was examined with 16S rRNA sequences, including an outgroup (*A. hydrophila*), and five sequences from environment, human, and fish isolates (Figure 2). The sequences showed clear clustering according to each isolated source, environment (red), human (green), and fish (blue), and the new isolate SKNUCL01 clustered with the environment group. The isolates were thus ultimately identified as *C. cucumeris*, and the 16S rRNA sequences were deposited in GenBank under the accession number MK280733.

### 2.4. Virulence Test of SKNUCL01

**Figure 3:**
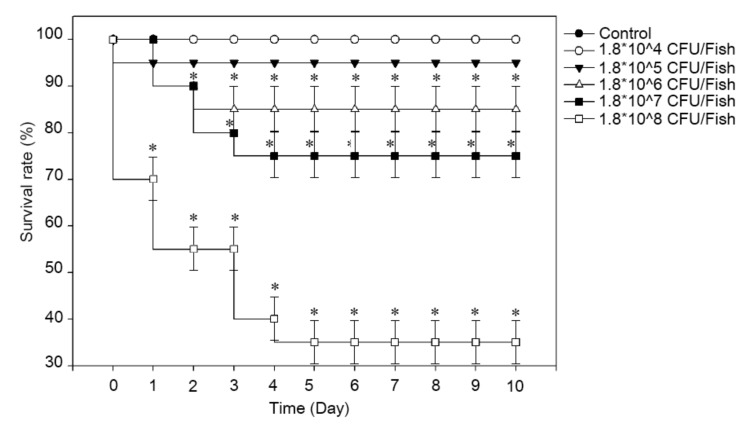
Pathogenicity of *C. cucumeris* SKNUCL01-challenged pond loach by intraperitoneal injection. Fish were infected with *C. cucumeris* SKNUCL01 at four different concentrations (10^5^: empty circle, 10^6^: filled triangle, 10^7^: empty triangle, and 10^8^: filled square). The fish injected with PBS served as the control group (filled circle). The mortality was recorded for a 10 day period after challenge. The values were presented as the mean from three independent experiments. The statistical significance was calculated using a one-way ANOVA with Bonferroni post-test.

The virulence was analyzed by challenging the fish with the isolated bacteria by immersion or injection. The challenge by immersion was conducted with or without artificial abrasion. Without abrasion, the challenged fish were not affected by *C. cucumeris* even when the exposure time was increased by 24 h. By contrast, with abrasion, the fish showed morbidity at 2 days post infection; mortality was detected after 5 days post infection, reaching up to 20% (4/20). As shown in Figure 3, an intraperitoneal challenge revealed 5% mortality for 10^5^ CFU/Fish (1/20; empty circle), 15% mortality for 10^6^ CFU/Fish (3/20; filled triangle), 25% mortality for 10^7^ CFU/Fish (5/20; empty triangle), and 65% mortality for 10^8^ CFU/Fish (13/20; filled square). The calculated LD_50_ was 8.52 × 10^7^ CFU/ml. During the observation, lethargy, loss of appetite, and abnormal swimming were recorded from the infected fish. To confirm Koch’s postulates, the bacteria were recovered from the liver and spleen as a pure isolate and identified as *C. cucumeris*. 

The biofilm-forming property, associated with *C. cucumeris* virulence was observed in a polystyrene 96-well plate for 48 h, and the optical density (OD) value was measured at 595 nm; this was found to be 2.18 ± 0.4 and 3.31 ± 0.34 at 24 and 48 h, respectively.

### 2.5. Antibiotic Susceptibility Test of SKNUCL01 

An antibiotic susceptibility test was conducted following the guideline of the Clinical and Laboratory Standards Institute (CLSI) M100; interpretive standards for other non-*Enterobacteriaceae* were used to determine the susceptibility of the isolated bacteria. *C. cucumeris* SKNUCL01 was resistant to ampicillin, ampicillin-sulbactam, cefotaxime, aztreonam, imipenem, meropenem, amikacin, gentamicin, ciprofloxacin, levofloxacin, and trimethoprim-sulfamethoxazole. It had intermediate resistance to piperacillin and minocycline and was susceptible to piperacillin-tazobactam, ceftazidime, and cefepime (Table 1). 

### 2.6. Phenotypic Screening for β-Lactamases 

To detect the β-lactamase that might affect the resistance of *C. cucumeris* SKNUCL01, phenotypic tests were performed using cephalosporins (ceftazidime, ceftriaxone, cefepime, and cefotaxime) and carbapenems (imipenem and meropenem). The combined disk test (CDT), double-disk synergy test (DDST), and modified Hodge test (MHT) were performed to detect metallo-beta lactamases (MBLs), serine penicillinases, and carbapenemases, respectively. In the CDT, cefotaxime and cefepime treatment showed a significant increase in the inhibition diameter, as shown in Table 2. In addition, the diameter increased in ceftriaxone (2 mm), meropenem (2 mm), and imipenem (3 mm). However, there were no changes when ceftazidime was combined with ethylenediaminetetraacetic acid (EDTA). In the DDST, co-treatment with amoxicillin-clavulanic acid showed no synergetic effect on the antibiotic disks examined (Table 2). Moreover, in the MHT, all of the results were negative for all antibiotics tested. 

### 2.7. Phenotypic Screening for Efflux Pump against Antibiotics 

An efflux pump inhibitor, phenylalanine-arginine β-naphthylamide (PAβN), did not inhibit the growth of *C. cucumeris* SKNUCL01 up to a concentration of 100 μg/mL (data not shown). The minimum inhibitory concentrations (MICs) of piperacillin and minocycline decreased upon treatment with 25 μg/mL PAβN. Further, 50 μg/mL of PAβN treatment resulted in a decrease in imipenem and minocycline MICs and a significant decrease in the piperacillin MIC. The MICs of piperacillin, imipenem, and minocycline were significantly decreased (by four-fold) and in the case of ceftazidime, the MIC was decreased by two-fold with the treatment of 100 μg/ml of PAβN (Table 3).

### 2.8. Genetic Screening for Antibiotic Resistance 

Antibiotic resistance-related genes such as β-lactamase and, efflux pumps associated with the phenotypic resistance of SKNUCL01 were screened for further elucidation of the resistance mechanism. Since SKNUCL01 showed a positive result only in the CDT test for MBL phenotypic detection, we performed genetic screening for MBL genes, such as *bla*_CIA_, *bla*_CGA_, *FOX*, *bla*_GES_, *bla*_GIM_, *bla*_IND_, *bla*_KPC_, *bla*_OXA_, and *bla*_VIM_. In total, nine β-lactamase and 15 efflux pump genes related to antibiotic resistance were examined (Table S2). *C. cucumeris* SKNUCL01, a multiple antibiotic resistant strain, did not harbor any of the genes.

## 3. Discussion

*Chryseobacterium cucumeris* SKNUCL01 was isolated from a diseased loach from a local aquaculture farm that showed gross skin infection signs, such as ulcers on their skin, loss of the epidermis, or fungus-like white patches. A yellowish colony with a distinct odor was isolated from the skin, liver, and spleen of the infected loach. Because of this distinct odor, *Chryseobacterium* spp. have often been considered as food spoilage bacteria [22,23,24]. Recently, however, *Chryseobacterium* originally recovered from food products have been isolated from diseased animals—*C. shigense* from trout and *C. oranimense* from humans [11,25]. Likewise, *C. cucumeris* was originally isolated from the cucumber plant (*Cucumis sativus*) [26]. Based on the VITEK 2 bacterial identification test, the isolated strain was found to share biochemical characteristics with *C. cucumeris*. The 16S rRNA gene sequence of the isolate most closely matched with *C. cucumeris* GSE06T (GenBank accession no. KX146463) in the EzBioCloud 16S database and was registered at GenBank under the accession number MK280733. This result suggests that the biochemical characteristics analyzed using VITEK 2 could be used to distinguish the *Chryseobacterium* genus at the species level.

Previously, from diseased loaches, bacterial diseases caused by *Flavobacterium columnare* [6], *Vibrio cholera* [7], *Aeromonas hydrophila* [8], *A. sobria* [9], *Listonella anguilarum* [27], and *Shewanella putrifacience* [27] have been reported; however, there has been no report of *Chryseobacterium* infection. There have been increasing reports of infections caused by this organism, which are related to diseased farmed fish, including *C. aahli* from lake trout [28], *C. indologenes* from yellow perch [29], and *C. piscicola* from salmonid fish [30]. The gross clinical signs caused by *Chryseobacterium* are ulcerative skin lesions and severe systemic infection. Similarly, the major clinical sign of the fish infected by *C. cucumeris* SKNUCL01 was skin ulcers, which presumably caused mortality.

A virulence study was conducted using intraperitoneal injection and bath immersion with or without artificial abrasion on the skin. The injection method revealed that the *C. cucumeris* SKNUCL01 strain, with an LD_50_ of 8.52 × 10^7^ CFU/ml, was pathogenic to the pond loach. In the immersion without abrasion method, neither mortality nor morbidity was detected; there was no increase in mortality even after prolonged exposure (~24 h) to bacteria. In contrast, immersion with artificial skin abrasion showed a 20% mortality at the concentration of 6 × 10^7^ CFU/ml after exposure to bacteria for 1 h. These results support the general observation of opportunistic pathogens that the infection could be encouraged by the abrasion on the fish body [31,32].

The exact virulence factor has not been fully explored for *Chryseobacterium*; however, the production of biofilm and protease were suggested as potential virulence factors [33]. *C. cucumeris* SKNUCL01 can form a biofilm at the air—liquid interface rather than on the side and bottom of the polystyrene plate (Figure S1). The OD_595_ value for detecting a biofilm was above 3.0 after 24 h of incubation; this value indicates that the bacterium is a strong biofilm former, based on the criteria of Snoussi et al. [34].

The major concern related to *Chryseobacterium* infection is its antibiotic resistance. The organisms isolated from human clinical sources are generally resistant to beta-lactams and carbapenems. Similarly, over 90% of the bacteria isolated from aquatic animals and the environment have developed resistance to polymyxin B, carbapenems (imipenem and meropenem), cefotaxime, amoxicillin-clavulanic acid, aztreonam, and tetracycline [21]. Another study concentrated on the resistance against widely used antibiotics, including chloramphenicol, florfenicol, and oxytetracycline, in aquaculture [20]. They detected that over 90% of the isolates were resistant to the above-mentioned three antibiotics; however, almost all of the isolates were susceptible to trimethoprime-sulphonamide. This same pattern was observed in the human clinical isolates through the SENTRY Antimicrobial Surveillance Program conducted from 1997 to 2001 [35]. More recently, trimethoprime-sulphonamide has been considered to be the most potent antibiotic against *Chryseobacterium* [15,36]. However, the *C. cucumeris* SKNUCL01 isolated in this study was only susceptible to piperacillin/tazobactam, ceftazidime, and cefepime, while being resistant to trimethoprim/sulfamethoxazole, levofloxacin, and ciprofloxacin, which were formerly considered as effective treatments. The antibiotic resistance of this strain can be explained by the activity of MBLs and the efflux pump. Through the CDT, the presence of MBLs was elucidated using EDTA, an MBL inhibitor, which shows a significant increase in the inhibition zone of cefotaxime and cefepime. In addition, the MICs of piperacillin, imipenem, minocycline, and ceftazidime against *C. cucumeris* SKNUCL01 could be decreased by treatment with the efflux pump inhibitor. Previous reports showed that the efflux pump is an antibiotic resistance mechanism in *Chryseobacterium* and *Flavobacterium*, a closely related organism to *Chryseobacterium*, isolated from fish [20,37]. Michel et al. [20] suggested that the antibiotic (phenicols) resistance of *Chryseobacterium* was related to the efflux pump. More recently, Clark et al. [37] reported the MIC of antibiotics other than phenicols, such as erythromycin, levofloxacin, linezolid, and norfloxacin, which can be affected by the efflux pump in the *Flavobacterium*. 

To elucidate the antibiotic resistance mechanism, we performed genetic screening. The *FOX*, bla_GES_, *bla*_IND_, *bla*_KPC_, *bla*_OXA-48_, and *bla*_VIM_ genes, which are well known for conferring resistance to carbapenems, were examined first [38,39,40,41,42,43,44]. Then, the *bla*_IND_ was screened for the presence of the enzyme that hydrolyzes the cephalosporins. However, the results were negative. Thus, we finally conducted screening for the *β*-lactamase genes previously reported from the *Chryseobacterium* genus such as *bla*_CIA_ [45], and *bla*_CGA_ [46], which showed negative results based on PCR. Further, we performed an efflux pump screen using the following primer sets: AcrAB-TolC set for tetracyclines or fluoroquinolones resistance; CmeABC set for tetracyclines; MexAB-OprD, MexCD-OprJ, and MexXY-OprN sets for *β*-lactams, fluoroquinolones, and tetracyclines. However, the genetic screening for the efflux pump also showed negative results as their activity did not exactly parallel those of the previously documented efflux pumps [47,48,49]. 

Using the inhibitors, we verified that MBLs and the efflux pump may be involved in the antibiotic resistance mechanism of *C. cucumeris* SKNUCL01. Inversely, this means that inhibitors such as EDTA, generally used for reducing heavy metals in aquaculture, could possibly be used as a synergistic agent of antibiotics. Although the antibiotic resistance-associated genes harbored by *C. cucumeris* SKNUCL01 have not been clearly proven, we focused on the characteristics of this organism as an opportunistic pathogen and provided clues against the multiple resistance *Chryseobacterium* outbreak.

## 4. Materials and Methods 

### 4.1. Ethical Statement

The procedures involving animals were approved by the Institutional Animal Care and Use Committee (IACUC) of Seoul National University (SNU-190228-2), and the experiments were performed in accordance with approved guidelines.

### 4.2. Bacterial Isolates and Culture Condition

About 50-day-old moribund loaches (*M. anguillicaudatus*) with suspected columnaris were observed in a local aquaculture farm located in Gyeong-gi province, South Korea. Fish that showed lethargic activity, loss of appetite, and skin infection were collected. The skin lesions showed ulceration, loss of the epidermis, and fungus-like white patches. The natural mortality was less than 1%; however, increased mortality was observed on the farm for several weeks, and the cumulative mortality surged to 10%. Some of these fish were brought to our aquatic-animal facility (Laboratory of Aquatic Biomedicine, Seoul National University) and divided into two groups: therapy and observation groups. The moribund fish in the therapy group were treated with oxytetracycline hydrochloride (50 mg/L) for 5 days, followed by erythromycin (25 mg/L) for 12 hours by the immersion method. Bacteria were recovered from the skin lesions, liver, and spleen of the observation group. The skin lesions on the diseased fish were washed with sterile phosphate-buffered saline to minimize contamination and inoculated on tryptic soy agar (TSA, BD). The surfaces of the liver and spleen were sterilized with a 70% alcohol swab, and the sections were inoculated on TSA. After an overnight culture (~18 h) at 25°C, yellow and shiny colonies with a distinct smell were predominantly observed on the culture plates. The colonies were randomly picked and streaked onto TSA. After recovering single colonies, the culture was stored in glycerol at −80°C. The bacterial strains were cultured on TSA at 25 °C.

### 4.3. Histological Analysis of the Skin Lesion

The fish showing severe skin ulceration and lethargic activity were sacrificed immediately. The normal and ulcerative skin samples were preserved and fixed in neutral-buffered formalin (10%), dehydrated with ethanol, and embedded in paraffin blocks. Then, the blocks were sectioned and stained using hematoxylin and eosin. The specimens were analyzed with light microscopy and digitally scanned by Xenos Inc. (Korea).

### 4.4. Identification of Isolated Bacterial Strains

To identify the isolates, Gram staining, biochemical tests using the VITEK 2 system, and 16S rRNA gene sequencing were performed. The gram staining was conducted with Color Gram 2 (bioMérieux, Craponne, France) following the manufacturer’s instructions. The isolated bacteria were subjected to biochemical identification using the VITEK 2 system. A single colony was suspended in 3 mL of 0.45% saline solution and the turbidity was adjusted to a 0.5 McFarland standard. The VITEK 2 GN card was then inoculated with the bacterial suspension. For the 16S rRNA gene analysis, the genomic DNA of the bacteria was extracted by a conventional heat method [50]. In brief, 1 mL of overnight bacterial cultures were centrifuged (12,000× *g*, 4 °C) and re-suspended in 100 μL of TE buffer, and then the solution was heated for 10 min at 100 °C and centrifuged again. The 16S rRNA gene was amplified with the universal primer set 27F/1492R using the supernatant [51]. The 16S rRNA gene was sequenced at Macrogen (Seoul, Korea), and the sequences obtained were compared to identify the bacterial strains using BLAST and EzTaxon [52]. The obtained partial 16S rRNA sequence (1466 bp) was deposited in GenBank under the accession number MK280733. Alignment was conducted and the phylogenetic tree was constructed using the maximum-likelihood method, implemented in MEGA7 [53]; 1000 bootstrap replications were used to build the phylogenetic tree.

### 4.5. Virulence of C. cucumeris SKNUCL01 in Pond Loach

To analyze the virulence of *C. cucumeris* SKNUCL01, a bath immersion with or without artificial abrasion and intraperitoneal (IP) injection was performed. In total, 500 healthy adult pond loaches (13–17 g) were obtained from another farm located in the Jeonbuk province, South Korea, and acclimated at 25 °C for 30 days before challenge in a recirculating aquaculture system. The fish were fed with commercial Tetra Bits Complete (Tetra, Germany) at 2% body weight daily. The fish were maintained in a 200 L fiber glass tank under a 12 h light/dark schedule. For the virulence study, 10 fish were randomly allocated per each group in the 2 L aquaria (20 × 45 cm).

For the immersion challenge, the overnight bacterial culture was inoculated with 1000 mL of water to adjust the density to 2.0 × 10^7^ CFU/mL and acclimated with aeration for 1 h. Then, the pond loaches were immersed in bacterial water and normal water (control) for 1 h and 24 h. For the artificial abrasion test, abrasion (1 × 0.5 cm) was introduced beside the pectoral fin by removing the skin, using a scalpel with anesthesia by tricaine methanesulfonate (500 mg/L; pH 7 adjusted by sodium bicarbonate). Then, the pond loaches were immersed in bacterial water and normal water (control) for 1 h. After immersion, the fish were returned to 2 L aquaria (10 fish per tank).

To calculate the LD_50_, each dilution of bacterial solution was injected into the pond loach via the intraperitoneal route, according to the Miller and Tainter method [54]. Four different doses (1.8 × 10^5^–1.8 × 10^8^ CFU/fish) and PBS (control group) were administered under anesthesia using tricaine methanesulfonate, and the fish were returned to the 2 L aquaria (10 fish per tank). The mortality was recorded daily for 10 days post-challenge. Dead fish were removed once daily, and the presence of *C. cucumeris* in the dead fish was confirmed by culturing spleen samples. 

At the end of the study, the fish were euthanized using tricaine methanesulfonate (2500 mg/L; pH 7 adjusted using sodium bicarbonate), and the rearing water was sterilized with bleach to prevent the spread of *C. cucumeris* SKNUCL01, a multidrug-resistant fish pathogen. 

### 4.6. Biofilm Formation of C. cucumeris SKNUCL01

To verify the biofilm-forming ability, *C. cucumeris* SKNUCL01 was cultured in 96-well polystyrene tissue culture microplates (Nunc, Roskilde, Denmark) without shaking, according to Snoussi et al. [34] with minor modifications. A 1% (v/v) overnight culture was inoculated into tryptic soy broth with or without glucose (1%), and then 200-μL aliquots were distributed into each well. The total biomass of the biofilm was quantified at 30 min (control), 24 h, and 48 h post-inoculation. The biofilms were stained with crystal violet solution (1%) for 15 min and washed with phosphate-buffered saline. Then, stained crystal violet was recovered in a 200 μL ethanol-acetone solution (80:20 v/v), and the OD was measured at 595 nm.

### 4.7. Antibiotic Susceptibility Test

The minimum inhibitory concentration (MIC) was determined using the VITEK AST-N212 susceptibility test card as per the manufacturer’s protocol. Briefly, 1 mL of overnight-grown bacteria in Mueller-Hinton broth (MHB; Becton Dickinson) was washed with sterile 0.45% sodium chloride solution. The suspension was added to 3 mL of sterile 0.45% sodium chloride solution to achieve McFarland No. 0.5. Then, the VITEK AST card was filled with the suspension. All the MICs were confirmed with three independent replicates. The antibiotic susceptibility profiles for ampicillin/sulbactam, piperacillin, piperacillin/tazobactam, cefotaxime, ceftazidime, cefepime, aztreonam, imipenem, meropenem, amikacin, gentamicin, ciprofloxacin, levofloxacin, minocycline, tigecycline, and trimethoprim/sulfamethoxazole were evaluated in accordance with the breakpoints suggested by the CLSI guidelines [55]. The susceptibility test was confirmed using *Escherichia coli* (ATCC 25922; antibiotic susceptible indicator).

### 4.8. Phenotypic Tests for Detection of β-Lactamases

To evaluate the antibiotic resistance mechanisms, phenotypic tests were performed to detect the β-lactamases that hydrolyze the cephalosporins (ceftazidime, ceftriaxone, cefepime, and cefotaxime) and carbapenems (imipenem and meropenem). The overnight-grown *C. cucumeris* SKNUCL01 was adjusted to a turbidity of McFarland No. 0.5 and inoculated on the Muller-Hinton agar plate using a sterile cotton swab. In the CDT, two sets of antibiotic disks (mentioned above), with one set containing 10 μL of 0.1 M EDTA, were placed 25 mm apart, center to center. A difference in the inhibition zone (≥4 mm, diameter) around the antibiotic EDTA disk compared to that of the antibiotic disk alone was interpreted as positive for an MBL [56]. In the DDST, amoxicillin-clavulanic acid disks (20/10 μg) were placed 27 mm, center to center, away from the above-mentioned antibiotic disks [57]. Enhancement of the inhibition zone by the β-lactam inhibitor was interpreted as positive. In the MHT, *E. coli* (ATCC 25922) adjusted to McFarland No. 0.5 was inoculated evenly on the Mueller-Hinton agar plate using a sterile cotton swab as described elsewhere [58]. After drying for 5 min, the above-mentioned set of antibiotic disks were placed at the center of the plate, and *C. cucumeris* SKNUCL01 was streaked heavily from the edge of the disk to the periphery of the plate. The zone of inhibition from the indicator strain was examined for a clover-like indentation following the growth of *C. cucumeris* SKNUCL01. All three tests were interpreted after 20 h of incubation at 37 °C.

### 4.9. Effect of PAβN on the MIC

The effect of PAβN, an efflux pump inhibitor, on the MICs was determined using the VITEK AST-N212 susceptibility test card. Briefly, 1 mL of overnight-grown bacteria in MHB was washed with sterile 0.45% sodium chloride solution. The suspension was added to 3 mL of sterile 0.45% sodium chloride solution containing 0, 25, 50, or 100 μg/mL PAβN (Sigma) to achieve McFarland No. 0.5. Then, the VITEK AST card was filled with the suspension. All the MICs were confirmed by three independent replicates. Four-fold or greater differences in MICs compared to the control were considered significant [37].

### 4.10. Detection of Antibiotic Resistance-Related Genes

To elucidate the antibiotic resistance mechanisms of *C. cucumeris* SKNUCL01, β-lactamase, and efflux pump genes were PCR screened. The DNA of the isolate was extracted as mentioned above. The presence of following genes—β-lactamases (*bla*_CIA-1_, *bla*_CGA-1_, *bla*_FOX_, *bla*_GES_, *bla*_GIM_, *bla*_IND_, *bla*_KPC_, *bla*_OXA-48_, *bla*_VIM_) and efflux pumps (AcrAB-TolC, CmeABC, MexAB-OprD, MexCD-OprJ, and MexXY-OprN)—were examined as described previously [39,40,41,42,43,44,45,46,47,48,49,50]. Shortly, the amplification was performed with 10 min of denaturation at 95 °C, followed by the 35 cycles of 1 min at 95 °C, 1 min at annealing temperature (Table S2), and 2 min at 72 °C and a 10 min of extension at 72 °C. All the primer sets used in this study are shown in Table S2.

### 4.11. Statistical Analyses

The one-way analysis of variance (ANOVA) with the Bonferroni post hoc test was performed to verify the statistical significance using SigmaPlot v12.0 (Systat Software, Inc. Chicago, IL, USA). The statistical significance was set at a P value under 0.05.

## 5. Conclusions

In this study, we provide the first description of the recovery of *C. cucumeris* from a diseased pond loach (*M. anguillicaudatus*), an important food and medicinal animal in East Asia. This pathogen could infect the host through a wound, which is characteristic of opportunistic pathogens. The resistance of *C. cucumeris* SKNUCL01 was significantly high to antibiotics formerly considered as effective treatments, such as trimethoprim/sulfamethoxazole, levofloxacin, and ciprofloxacin. Moreover, the isolate was resistant to most of the third-generation cephalosporins and carbapenems tested. We further showed that MBLs and the efflux pump affect the antibiotic resistance of this strain. Although genetic studies are insufficient to clearly elucidate the antibiotic resistance mechanisms, EDTA or PAβN could increase bacterial susceptibility to several antibiotics and thus provide a breakthrough for the aquaculture industry against multidrug-resistant *Chryseobacterium* infection. 

## Figures and Tables

**Table 1:** Antimicrobial susceptibility profiles of *C. cucumeris* SKNUCL01 by minimal inhibitory concentration.

Antimicrobial Agent	Interpretation Range ^a^			MIC ^c^
	S ^b^	I	R	
Piperacillin	≤16	32–64	≥128	64
Piperacillin/ tazobactam	≤16/4	32/4–64/4	≥128/4	≤4/4
Ceftazidime	≤8	16	≥32	4
Cefepime	≤8	16	≥32	≤1
Cefotaxime	≤8	16-32	≥64	≥64
Aztreonam	≤8	16	≥32	≥64
Imipenem	≤4	8	≥16	≥16
Meropenem	≤4	8	≥16	≥16
Amikacin	≤16	32	≥64	≥64
Gentamicin	≤4	8	≥16	≥16
Minocycline	≤4	8	≥16	8
Ciprofloxacin	≤1	2	≥4	≥4
Levofloxacin	≤2	4	≥8	≥8
Trimethoprim/ sulfamethoxazole	≤2/38	-	≥4/76	≥4/76

^a^ The interpretative criteria for other non-*Enterobacteriaceae* suggested by Clinical and Laboratory Standards Institute (CLSI) were used. ^b^ S: sensitive; I: intermediate resistant; R: resistant. ^c^ MIC: minimum inhibitory concentration (μg/mL).

**Table 2:** Phenotypic tests for the detection of β-lactamases. Combined disk test (CDT), double-disk synergy test (DDST), and modified Hodge test (MHT) were performed to detect the metallo-beta-lactamase, serine penicillinases, and carbapenemases.

	CDT ^a^	DDST ^a^	MHT ^b^
**Ceftazidime**	0	0	-
**Ceftriaxone**	3	0	-
**Cefepime**	8	0	-
**Cefotaxime**	18	0	-
**Imipenem**	3	0	-
**meropenem**	2	0	-

**^a^** The results were analyzed in the increased diameter (mm) of the inhibition zone. **^b^** The results were analyzed in the margin of the inhibition zone. Negative (-): circle shape; positive (+): clover shape.

**Table 3:** Effect of phenylalanine-arginine β-naphthylamide (PAβN) on the antimicrobial susceptibility profiles of *C. cucumeris* SKNUCL01.

Antimicrobial Agents	MIC			
	Control	PAβN 25 μg/mL	PAβN 50 μg/mL	PAβN 100 μg/mL
Piperacillin	64	32	16 *	8 *
Piperacillin/tazobactam	≤4/4	≤4/4	≤4/4	≤4/4
Ceftazidime	4	4	4	2
Cefepime	≤1	≤1	≤1	≤1
Cefotaxime	≥64	≥64	≥64	≥64
Aztreonam	≥64	≥64	≥64	≥64
Imipenem	≥16	≥16	8	4 *
Meropenem	≥16	≥16	≥16	≥16
Amikacin	≥64	≥64	≥64	≥64
Gentamicin	≥16	≥16	≥16	≥16
Minocycline	8	4	4	2 *
Ciprofloxacin	≥4	≥4	≥4	≥4
Levofloxacin	≥8	≥8	≥8	≥8
Trimethoprim/sulfamethoxazole	≥4/76	≥4/76	≥4/76	≥4/76

* More than four-fold minimum inhibitory concentration (MIC) decrease.

## Supplementary Materials

The following are available online at https://www.mdpi.com/2076-0817/9/5/397/s1: Table S1: biochemical profiles of *C. cucumeris* SKNUCL01 were compared with type strains. Table S2: primer sets and PCR conditions used in this study.

## References

[1] Saito K. (1988). Movement and spawning of several freshwater fishes in temporary waters around paddy fields. Jpn. J. Ecol..

[2] Kim J.O., Shin H.S., Yoo J.H., Lee S.H., Jang K.S., Kim B.C. (2011). Functional evaluation of small-scale pond at paddy field as a shelter for mudfish during midsummer drainage period. Korean J. Environ. Biol..

[3] Zhang H., Lu X., Zhang Y., Ma X., Wang S., Ni Y., Chen J. (2016). Bioaccumulation of organochlorine pesticides and polychlorinated biphenyls by loaches living in rice paddy fields of Northeast China. Environ. Pollut..

[4] You L., Zhao M., Regenstein J.M., Ren J. (2011). *In vitro* antioxidant activity and *in vivo* anti-fatigue effect of loach (*Misgurnus anguillicaudatus*) peptides prepared by papain digestion. Food Chem..

[5] You L., Zhao M., Liu R.H., Regenstein J.M. (2011). Antioxidant and antiproliferative activities of loach (*Misgurnus anguillicaudatus*) peptides prepared by papain digestion. J. Agric. Food Chem..

[6] Chowdhury M.B.R., Wakabayashi H. (1991). A study on *Flexibacter columnaris* infection in loach, *Misgurnus anguillicaudatus* (Bleeker, Günther). J. Fish Dis..

[7] Zhang X.J., Yao D.R., Yan B.L., Bi K.R., Liang L.G., Qin G.M. (2012). Identification of *Vibrio cholerae* as a causative bacterium for an ulcer disease of cultured loach *Misgurnus anguillicaudatus* in China. Afr. J. Microbiol. Res..

[8] Jun J.W., Kim J.H., Gomez D.K., Choresca C.H., Han J.E., Shin S.P., Park S.C. (2010). Occurrence of tetracycline-resistant *Aeromonas hydrophila* infection in Korean cyprinid loach (*Misgurnus anguillicaudatus*). Afr. J. Microbiol. Res..

[9] Zhu M., Wang X.R., Li J., Li G.Y., Liu Z.P., Mo Z.L. (2016). Identification and virulence properties of *Aeromonas veronii* bv. sobria isolates causing an ulcerative syndrome of loach Misgurnus anguillicaudatus. J. Fish Dis..

[10] Campbell S., Harada R.M., Li Q.X. (2008). *Chryseobacterium arothri* sp. nov., isolated from the kidneys of a pufferfish. Int. J. Syst. Evol. Microbiol..

[11] Zamora L., Vela A.I., Palacios M.A., Domínguez L., Fernández-Garayzábal J.F. (2012). First isolation and characterization of *Chryseobacterium shigense* from rainbow trout. BMC Vet. Res..

[12] Shahi N., Sharma P., Pandey J., Bisht I., Mallik S.K. (2018). Characterization and pathogenicity study of *Chryseobacterium scophthalmum* recovered from gill lesions of diseased golden mahseer, *Tor putitora* (Hamilton, 1822) in India. Aquaculture.

[13] Bernardet J.F., Vancanneyt M., Matte-Tailliez O., Grisez L., Tailliez P., Bizet C., Nowakowskie M., Kerouaulta B., Swings J. (2005). Polyphasic study of *Chryseobacterium* strains isolated from diseased aquatic animals. Syst. Appl. Microbiol..

[14] Chang Y.C., Lo H.H., Hsieh H.Y., Chang S.M. (2015). Identification, epidemiological relatedness, and biofilm formation of clinical *Chryseobacterium indologenes* isolates from central Taiwan. J. Microbiol. Immunol. Infect..

[15] Lambiase A., Del Pezzo M., Raia V., Sepe A., Ferri P., Rossano F. (2007). *Chryseobacterium* respiratory tract infections in patients with cystic fibrosis. J. Infect..

[16] Alfouzan W., Dhar R., Al-Hashemi H., Al-Sweih N., Albert M.J. (2014). Clinical and microbiological characteristics of Chryseobacterium spp. isolated from neonates in Kuwait. JMM Case Rep..

[17] Lin Y.T., Jeng Y.Y., Lin M.L., Yu K.W., Wang F.D., Liu C.Y. (2010). Clinical and microbiological characteristics of *Chryseobacterium indologenes* bacteremia. J. Microbiol. Immunol. Infect..

[18] Omar A., Camara M., Fall S., Ngom-Cisse S., Fall B., Ba-Diallo A., Diop-Ndiaye H., Toure-Kane C., Mboup S., Gaye-Diallo A. (2014). *Chryseobacterium indologenes* in a woman with acute leukemia in Senegal: A case report. J. Med. Case Rep..

[19] Didinen B.I., Onuk E.E., Öztürk T., Metin S., Meryem Ö.Z., Çayli Ö., Kubilay A. (2016). First report of Chryseobacterium sp. from Koi (Cyprinus carpio) in Turkey. Isr. J. Aquacult.-Bamid.

[20] Michel C., Matte-Tailliez O., Kerouault B., Bernardet J.F. (2005). Resistance pattern and assessment of phenicol agents’ minimum inhibitory concentration in multiple drug resistant *Chryseobacterium* isolates from fish and aquatic habitats. J. Appl. Microbiol..

[21] Maravić A., Skočibušić M., Šamanić I., Puizina J. (2013). Profile and multidrug resistance determinants of *Chryseobacterium indologenes* from seawater and marine fauna. World J. Microbiol. Biotechnol..

[22] Mielmann A. (2006). Food spoilage characteristics of *Chryseobacterium* species. Master’s Thesis.

[23] Bekker A. (2011). Growth and spoilage characteristics of *Chryseobacterium* species in milk. Master’s Thesis.

[24] Zheng L., Bae Y.M., Jung K.S., Heu S., Lee S.Y. (2013). Antimicrobial activity of natural antimicrobial substances against spoilage bacteria isolated from fresh produce. Food Control.

[25] Sharma P., Gupta S.K., Diene S.M., Rolain J.M. (2015). Whole-genome sequence of *Chryseobacterium oranimense*, a colistin-resistant bacterium isolated from a cystic fibrosis patient in France. Antimicrob. Agents Chemother..

[26] Jeong J.J., Lee D.W., Park B., Sang M.K., Choi I.G., Kim K.D. (2017). *Chryseobacterium cucumeris* sp. nov., an endophyte isolated from cucumber (*Cucumis sativus L*.) root, and emended description of *Chryseobacterium arthrosphaerae*. Int. J. Syst. Evol. Microbiol..

[27] Qin L., Zhu M., Xu J. (2014). First report of *Shewanella* sp. and *Listonella* sp. infection in freshwater cultured loach, *Misgurnus anguillicaudatus*. Aquac. Res..

[28] Loch T.P., Faisal M. (2014). Chryseobacterium aahli sp. nov., isolated from lake trout (Salvelinus namaycush) and brown trout (Salmo trutta), and emended descriptions of Chryseobacterium ginsenosidimutans and Chryseobacterium gregarium. Int. J. Syst. Evol. Microbiol..

[29] Pridgeon J.W., Klesius P.H., Garcia J.C. (2013). Identification and virulence of *Chryseobacterium indologenes* isolated from diseased yellow perch (*Perca flavescens*). J. Appl. Microbiol..

[30] Ilardi P., Fernandez J., Avendano-Herrera R. (2009). *Chryseobacterium piscicola* sp. nov., isolated from diseased salmonid fish. Int. J. Syst. Evol. Microbiol..

[31] Menanteau-Ledouble S., Karsi A., Lawrence M.L. (2011). Importance of skin abrasion as a primary site of adhesion for *Edwardsiella ictaluri* and impact on invasion and systematic infection in channel catfish *Ictalurus punctatus*. Vet. Microbiol..

[32] Abraham T.J., Sarker S., Dash G., Patra A., Adikesavalu H. (2017). *Chryseobacterium* sp. PLI2 and *Aeromonas hydrophila* co-infection in pacu, *Piaractus brachypomus* (Cuvier, 1817) fries cultured in West Bengal, India. Aquaculture.

[33] Pan H.J., Teng L.J., Chen Y.C., Hsueh P.R., Yang P.C., Ho S.W., Luh K.T. (2000). High protease activity of *Chryseobacterium indologenes* isolates associated with invasive infection. J. Mcrobiol. Immunol. Infect..

[34] Snoussi M.I., Noumi E., Cheriaa J., Usai D., Sechi L.A., Zanetti S., Bakhrouf A. (2008). Adhesive properties of environmental *Vibrio alginolyticus* strains to biotic and abiotic surfaces. New Microbiol..

[35] Kirby J.T., Sader H.S., Walsh T.R., Jones R.N. (2004). Antimicrobial susceptibility and epidemiology of a worldwide collection of *Chryseobacterium* spp.: Report from the SENTRY Antimicrobial Surveillance Program (1997–2001). J. Clin. Microbiol..

[36] Chen F.L., Wang G.C., Teng S.O., Ou T.Y., Yu F.L., Lee W.S. (2013). Clinical and epidemiological features of *Chryseobacterium indologenes* infections: Analysis of 215 cases. J. Mcrobiol. Immunol. Infect..

[37] Clark S.E., Jude B.A., Danner G.R., Fekete F.A. (2009). Identification of a multidrug efflux pump in *Flavobacterium johnsoniae*. Vet. Res..

[38] Barguigua A., El Otmani F., Talmi M., Reguig A., Jamali L., Zerouali K., Timinouni M. (2013). Prevalence and genotypic analysis of plasmid-mediated β-lactamases among urinary *Klebsiella pneumoniae* isolates in Moroccan community. J. Antibiot. Res..

[39] Poirel L., Le Thomas I., Naas T., Karim A., Nordmann P. (2000). Biochemical sequence analyses of GES-1, a novel class A extended-spectrum β-lactamase, and the class 1 integron In52 from *Klebsiella pneumoniae*. Antimicrob. Agents Chemother..

[40] Mendes R.E., Kiyota K.A., Monteiro J., Castanheira M., Andrade S.S., Gales A.C., Pignatari A.C.C., Tufik S. (2007). Rapid detection and identification of metallo-β-lactamase-encoding genes by multiplex real-time PCR assay and melt curve analysis. J. Clin. Microbiol..

[41] Bellais S., Poirel L., Leotard S., Naas T., Nordmann P. (2000). Genetic diversity of carbapenem-hydrolyzing metallo-β-lactamases from *Chryseobacterium (Flavobacterium) indologenes*. Antimicrob. Agents Chemother..

[42] Wolter D.J., Khalaf N., Robledo I.E., Vázquez G.J., Santé M.I., Aquino E.E., Goering R.V., Hanson N.D. (2009). Surveillance of carbapenem-resistant *Pseudomonas aeruginosa* isolates from Puerto Rican medical center hospitals: Dissemination of KPC and IMP-18 β-lactamases. Antimicrob. Agents Chemother..

[43] Beyrouthy R., Robin F., Cougnoux A., Dalmasso G., Darfeuille-Michaud A., Mallat H., Dabboussi F., Hamzé M., Bonnet R. (2013). Chromosome-mediated OXA-48 carbapenemase in highly virulent *Escherichia coli*. J. Antimicrob. Chemother..

[44] Monteiro J., Widen R.H., Pignatari A.C., Kubasek C., Silbert S. (2012). Rapid detection of carbapenemase genes by multiplex real-time PCR. J. Antimicrob. Chemother..

[45] Matsumoto T., Nagata M., Ishimine N., Kawasaki K., Yamauchi K., Hidaka E., Kasuga E., Horiuchi K., Oana K., Kawakami Y. (2012). Characterization of CIA-1, an Ambler class A extended-spectrum β-lactamase from *Chryseobacterium indologenes*. Antimicrob. Agents Chemother..

[46] Bellais S., Naas T., Nordmann P. (2002). Molecular and biochemical characterization of Ambler class A extended-spectrum β-lactamase CGA-1 from *Chryseobacterium gleum*. Antimicrob. Agents Chemother..

[47] Swick M.C., Morgan-Linnell S.K., Carlson K.M., Zechiedrich L. (2011). Expression of multidrug efflux pump genes acrAB-tolC, mdfA, and norE in *Escherichia coli* clinical isolates as a function of fluoroquinolone and multidrug resistance. Antimicrob. Agents Chemother..

[48] Olah P.A., Doetkott C., Fakhr M.K., Logue C.M. (2006). Prevalence of the *Campylobacter* multi-drug efflux pump (CmeABC) in *Campylobacter* spp. isolated from freshly processed turkeys. Food Microbiol..

[49] Murugan N., Malathi J., Therese K.L., Madhavan H.N. (2018). Application of six multiplex PCR’s among 200 clinical isolates of *Pseudomonas aeruginosa* for the detection of 20 drug resistance encoding genes. Kaohsiung J. Med. Sci..

[50] Dweba C.C., Zishiri O.T., El Zowalaty M.E. (2019). Isolation and molecular identification of virulence, antimicrobial and heavy metal resistance genes in livestock-associated methicillin-resistant *Staphylococcus aureus*. Pathogens.

[51] Lane D.J., Stackebrandt E., Goodfellow M. (1991). 16S/23S rRNA sequencing. Nucleic Acid Techniques in Bacterial Systematics.

[52] Chun J., Lee J.H., Jung Y., Kim M., Kim S., Kim B.K., Lim Y.W. (2007). EzTaxon: A web-based tool for the identification of prokaryotes based on 16S ribosomal RNA gene sequences. Int. J. Syst. Evol. Microbiol..

[53] Kumar S., Stecher G., Tamura K. (2016). MEGA7: Molecular evolutionary genetics analysis version 7.0 for bigger datasets. Mol. Biol. Evol..

[54] Miller L.C., Tainter M. (1944). Estimation of the ED50 and its error by means of logarithmic-probit graph paper. Proc. Soc. Exp. Biol. Med..

[55] Clinical and Laboratory Standards Institute (2014). Performance Standards for Antimicrobial Susceptibility Testing: Twenty-Fourth Informational Supplement, M100-S24.

[56] Franklin C., Liolios L., Peleg A.Y. (2006). Phenotypic detection of carbapenem-susceptible metallo-β-lactamase-producing gram-negative bacilli in the clinical laboratory. J. Clin. Microbiol..

[57] Polsfuss S., Bloemberg G.V., Giger J., Meyer V., Böttger E.C., Hombach M. (2011). Practical approach for reliable detection of AmpC beta-lactamase-producing *Enterobacteriaceae*. J. Clin. Microbiol..

[58] Lee K., Kim C.K., Yong D., Jeong S.H., Yum J.H., Seo Y.H., Docquier J.D., Chong Y. (2010). Improved performance of the modified Hodge test with MacConkey agar for screening carbapenemase-producing Gram-negative bacilli. J. Microbiol. Methods.

